# Factors affecting clinic attendance for children referred with middle ear conditions

**DOI:** 10.1111/ans.18140

**Published:** 2022-11-24

**Authors:** Holly McLaren, Raymond Kim, Wiremu MacFater, Michel Neeff, Tanja Jelicic, Richard Douglas

**Affiliations:** ^1^ Department of Surgery The University of Auckland Auckland New Zealand; ^2^ Department of Paediatric Otolaryngology Starship Children's Hospital Auckland New Zealand

**Keywords:** cholesteatoma, middle ear, otitis media, otitis media, otitis media with effusion, suppurative, tympanic membrane perforation

## Abstract

**Background:**

Middle ear disease is common in children and, if untreated, can lead to long term complications. This study investigated whether ethnic or socioeconomic inequities existed among children referred to a tertiary regional paediatric ORL service.

**Methods:**

All middle ear pathology related referrals to Starship Children's Hospital ORL service during 2018 and 2019 were reviewed. Online clinical records were accessed to collect demographic data and appointment outcomes. The relationships between ethnicity, socioeconomic status, age at referral and clinic attendance were analysed.

**Results:**

A total of 1530 children were referred and 214 (14.0%) of these did not attend their scheduled appointments. Compared with the age of referral for European children, Māori children were referred on average 1.1 years older while Pasifika children were referred 2.4 years older. Compared to European children, Māori children were 4.3 times more likely to miss an appointment while Pasifika were 7.2 times more likely. Socioeconomic status had no significant effect on either age of referral or clinic attendance.

**Conclusions:**

Māori and Pasifika children with middle ear disease are referred later and are more likely to miss outpatient clinic appointments. Pasifika children are affected more than Māori.

## Graphical abstract

Middle ear disease is common in children and can lead to long term complications. This article shows that Maori and Pasifika children with middle ear conditions are referred to paediatric ORL at a later age and are more likely to miss their clinic appointments. Socioeconomic status was not found to be a factor.

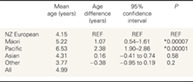

## Introduction

Between 50% and 85% of children will have had at least one episode of otitis media before the age of three.^1^ Children with recurrent otitis media or chronic otitis media with effusion (OME) may require a surgical intervention such as insertion of tympanostomy tubes. Delays in accessing appropriate specialist management can result in hearing loss, speech delay, mastoiditis or even intracranial infection.^2^ Insertion of tympanostomy tubes at a young age without delay has also been shown to reduce the incidence of cholesteatoma.^3^

In New Zealand, people of Māori or Pasifika ethnicities and those with lower socioeconomic status tend to have poorer health over a range of measures.^4^ One contributing factor is believed to be less access to healthcare. Analysis of national datasets show that Maori and Pasifika children are less likely to be brought to their outpatient ORL clinic appointments, and perhaps accordingly, rates of acute hospital admission with complications of otitis media are higher.^5^ However, few data have been published on the extent that these disparities apply to children with middle ear disease.

From referral to definitive management of middle ear disease, there are several points at which inequities could exist. This study aimed primarily to evaluate whether socioeconomic or ethnic differences exist in the age of referral of children with middle ear disease and whether these differences influence the rate of non‐attendance to outpatient clinic appointments. The appointment outcomes of the children who did not attend were then recorded. We set out to identify where inequities exist in our healthcare system, thereby allowing subsequent reduction of these inequities.

## Methods

### Ethics

Approval for this study was obtained from the Auckland Health Research Ethics Committee (AHREC).

### Location

Starship Children's Hospital is a tertiary paediatric centre in Auckland, New Zealand. The paediatric otolaryngology department serves the central Auckland region as well as a number of children referred from other regions in New Zealand and from Pacific nations.

### Patient selection

All referrals to Starship Children's Hospital paediatric otolaryngology in 2018 and 2019 were identified. These were each read to determine the reason for referral. Those categorized as ‘middle ear disease’ included referrals for assessment of recurrent otitis media, otitis media with effusion (OME), chronic suppurative otitis media (CSOM) and tympanic membrane perforation. Cerumen impaction was not included. Referrals for children from outside of New Zealand were excluded.

### Data collection and analysis

Electronic clinical records were retrospectively reviewed. Ethnicity was grouped into New Zealand European, Maori, Pasifika, Asian and Other. Ethnicity data were compared with the New Zealand Census data from the same time period. Gender and age at referral were recorded. Socioeconomic status was determined using the New Zealand Deprivation Index^6^ which is calculated using the home address. Index deciles ranged from 1 (least deprived) through 10 (most deprived).

Attendance or non‐attendance to the first outpatient clinic appointment was recorded. For children who did not attend, clinical records were individually examined. The appointment outcome was recorded as rescheduled or discharged. If discharged, it was recorded if a subsequent referral was made. If rescheduled, attendance to the subsequent appointment was also noted.

### Statistical analysis

The data were analysed using R software.^7^ The relationship of demographic variables to attendance (a binomial variable) was analysed using a chi‐squared test and a logistic regression model. Simple linear regression was used to assess relationships of demographic variables with age at referral being a continuous variable. Finally, a multiple regression analysis was performed to compare association between decile, age at referral, attendance and ethnicity. Results were considered statistically significant when the *P*‐value was less than 0.05.

## Results

A total of 4679 individual children were referred in 2018 and 2019. Of these, 1530 children (32.6%) were referred with middle ear disease. A total of 4 were excluded from the final analysis as they lived outside of New Zealand in Pacific countries. The final participant population comprised of 1526 children.

Over this period, 214 children (14.0%) did not attend their first scheduled outpatient clinic appointment: 21 cancelled their appointment ahead of time, 9 opted to be seen in private and 1 patient was deceased (unrelated to middle ear pathology). All other children were recorded as having attended their allocated appointment.

### Demographics

Table 1 summarizes the demographic distribution of the study group. Age at referral is shown in Table 2 and ranged from zero to 19. Four were aged 16 or older. Ethnic the Pasifika group was mostly made up of Samoan (*n* = 157, 37.3%) and Tongan (*n* = 153, 36%). Asian ethnicity group was mostly Indian (*n* = 104, 44%) and Chinese (*n* = 58, 25%). The Other ethnicity group was predominantly ‘Other European’ (*n* = 91, 65%) Table 3.

**Table 1:** Demographics of children referred to paediatric ORL with middle ear disease

	Ethnicity: *n* (%)					
	NZ European	Māori	Pacific	Asian	Other	Total
Ethnicity	451 (30)	283 (18)	420 (28)	232 (15)	140 (9)	1526
Gender						
Male	267 (59)	151 (53)	257 (61)	122 (53)	79 (56)	876 (57)
Female	184 (41)	132 (47)	163 (39)	110 (47)	61 (44)	650 (43)
Decile^†^						
1–2	112 (25)	21 (8)	8 (2)	32 (14)	33 (24)	206 (13)
3–4	121 (27)	34 (12)	35 (8)	43 (19)	46 (33)	279 (18)
5–6	97 (21)	49 (17)	50 (12)	61 (26)	31 (22)	288 (19)
7–8	71 (16)	57 (20)	96 (23)	49 (21)	14 (10)	287 (19)
9–10	50 (11)	122 (43)	231 (55)	47 (20)	16 (11)	466 (31)

^†^ New Zealand Deprivation Index Decile

**Table 2:** Age of referral by ethnic group

	Mean age (years)	Age difference (years)	95% confidence interval	*P*
NZ European	4.15	REF	REF	REF
Māori	5.22	1.07	0.54–1.61	0.00007
Pacific	6.53	2.38	1.90–2.86	0.00001
Asian	4.31	0.16	−0.41 to 0.74	0.58
Other	3.77	−0.38	−0.95 to 0.19	0.2
All	4.99			

**Table 3:** Rate of non‐attendance to outpatient clinic by ethnicity

	% of total referred	OR	95% confidence interval	*P*
NZ European	4.9	REF	REF	REF
Maori	18	4.29	2.54–7.25	0.00001
Pacific	26.9	7.18	4.44–11.60	0.00001
Asian	9.1	1.94	1.04–3.61	0.036
Other	5	1.03	0.43–2.46	0.95

### Socioeconomic status

Univariate analysis was performed with socioeconomic decile and non‐attendance. A linear regression model showed a significant association between decile and non‐attendance, with an intercept of 6.1 (CI 5.9–6.2, *P* 0.00001) and regression coefficient of 1.3 for those who did not attend (CI 0.9–1.8, *P* = 0.00001). This means those who did not attend had a decile scale score of 1.3 units higher than those who did. Age at referral was then included showing a very small but significant association, with a regression coefficient of 0.1 (decile 0.1 units) higher for every year of age (CI 0.1–0.2, *P* = 0.00001).

Finally, ethnicity was integrated into the model. Compared with European children (intercept of decile 4.5, CI 4.2–4.8, *P* = 0.00001), those of Asian ethnicity had a regression coefficient of 1.1 (CI 0.7–1.5, *P* = 0.00001). This is interpreted as a statistically significant score of +1.1 units higher on the decile scale than Europeans. Likewise, the regression coefficient for Māori was 2.3 (CI 1.9–2.7, *P* = 0.00001) and for Pasifika was 3.1 (CI 2.7–3.4, *P* = 0.00001). The coefficient for the Other Ethnicity group was −0.2 decile scale units but this was not statistically significant (CI −0.7 to 0.2, *P* = 0.3).

When ethnicity was included in analysis, the effect of decile on attendance was no longer significant (coefficient of 0.3, CI −0.1 to 0.7, *P* = 0.1). The effect of decile on age at referral became even smaller, with a coefficient of only 0.06 decile units for each additional year of age (CI 0.02–0.09, *P* = 0.001).

### Appointment rescheduling

A total of 119 (55.6%) of appointments not attended were rescheduled and 88 (73.9%) of these children attended their next appointment. Of the 31 children who did not attend two appointments in a row, 13 were Māori and 15 were Pasifika (when combined, 90%). The percentage of children rescheduled was similar across ethnicities. 11 (50.0%) NZ European children, 32 (61%) Māori, 57 (50%) Pasifika, 15 (66%) Asian and 4 (55%) children classified as ‘Other’ ethnicity were rescheduled. Of the 95 children who were discharged rather than rescheduled, 39 (41%) were re‐referred.

## Discussion

We found significant ethnic disparities in both age of referral to paediatric ORL and appointment attendance. Māori children are referred more than a year later than children of other ethnicities. Pasifika children are referred over 2 years later. There are also differences in rates of non‐attendance to first outpatient clinic appointments. Compared with NZ European children, Māori children are more than four times as likely not to attend their first appointment, while Pasifika children are more than seven times as likely. Māori and Pasifika make up the majority (90%) of the small group of children who were not brought to two consecutive appointments.

Several previous studies have demonstrated higher rates of hospital non‐attendance for Māori and Pasifika: non‐attendance is between two and three times more common for both Māori and Pasifika, children^5^ and adults.^8^, ^9^

Middle ear disease symptoms such as hearing loss or discharging ear can sometimes go unrecognized as abnormal or be tolerated over a long time.^10^ Chronicity of symptoms may amplify pre‐existing difficulties in accessing treatment.

Language barrier may be a contributing factor, particularly for Pasifika families.

The age of referral has not previously been studied in this population. For children with middle ear disease, a delay of a year or more in accessing appropriate treatment may increase the risk of complications.^2^, ^3^ Reasons for referral delay are poorly understood and likely multifactorial, as referrals can follow several different pathways. Many younger children are referred by their general practitioner and some by community ear nurses who attend schools. Those who fail before school hearing screening tests at age four or five are assessed by audiologists and then referred. Difficulty in accessing primary care may be a significant reason why some children are not referred before age 5 years.

Interestingly, there was not any significant association between socioeconomic status (NZDep decile) and age of referral or rate of non‐attendance. Previously published studies have shown a variable relationship between deprivation index and middle ear disease: hospitalization rates for otitis media are higher for children living in low decile areas,^11^ while it was not found to be a risk factor for developing chronic OME.^12^

There were no ethnic differences in the rate of rescheduling missed appointments. The decision to reschedule is made by the clinician with whom the child was booked to see in the clinic. Most children (74%) were brought to their rescheduled appointment. This suggests that simply rebooking all missed first appointments could substantially reduce loss to follow up for children who do not attend initially. For the small number of children who miss two or more appointments (in this study 31 over 2 years), targeted interventions may be useful. In our hospital, Pasifika and Māori specific ‘Health Navigators’ are being deployed in the outpatient clinic setting. These are nurses who contact and support families prior to their appointments in order to help them attend.

This study did not include children who were referred to private ORL services. We do not know the number of children or the demographic distribution of this group in comparison to those referred to public services.

This study did not investigate reasons why children were not brought to appointments, and therefore the factors contributing to ethnic differences in attendance remain a matter for conjecture. Further research is required to determine how best to address these inequities.

We did not investigate long‐term complication rates of middle ear disease given the study period was only 2 years. As there is limited literature on this in New Zealand, it would be helpful to quantify the long‐term effects of delayed or missed referral.

In conclusion, Māori and Pasifika children with middle ear disease are referred to paediatric ORL services at a later age and they are also more likely to miss their outpatient clinic appointments. Pasifika children are affected more than Māori children. Delays in accessing appropriate treatment could contribute to higher complication rates in these ethnic groups.

## Author contributions

**Holly Mclaren:** Conceptualization; data curation; formal analysis; investigation; methodology; writing – original draft. **Raymond Kim:** Conceptualization; methodology; supervision; writing – review and editing. **Wiremu MacFater:** Conceptualization; methodology; writing – review and editing. **Michel Neeff:** Conceptualization; methodology; writing – review and editing. **Tanja Jelicic:** Conceptualization; methodology; writing – review and editing. **Richard Douglas:** Conceptualization; methodology; supervision; writing – review and editing.

## Conflict of interest

None declared.
